# The effects of eccentricity on attentional capture

**DOI:** 10.3758/s13414-023-02735-z

**Published:** 2023-05-31

**Authors:** Elle van Heusden, Christian N. L. Olivers, Mieke Donk

**Affiliations:** Faculty of Behavioral and Movement Sciences, Cognitive Psychology, Vrije Universiteit Amsterdam, Van der Boechorststraat 7, 1081 HV Amsterdam, The Netherlands

**Keywords:** Eye movements, Attentional capture, Visual search

## Abstract

Visual attention may be captured by an irrelevant yet salient distractor, thereby slowing search for a relevant target. This phenomenon has been widely studied using the additional singleton paradigm in which search items are typically all presented at one and the same eccentricity. Yet, differences in eccentricity may well bias the competition between target and distractor. Here we investigate how attentional capture is affected by the relative eccentricities of a target and a distractor. Participants searched for a shape-defined target in a grid of homogeneous nontargets of the same color. On 75% of trials, one of the nontarget items was replaced by a salient color-defined distractor. Crucially, target and distractor eccentricities were independently manipulated across three levels of eccentricity (i.e., near, middle, and far). Replicating previous work, we show that the presence of a distractor slows down search. Interestingly, capture as measured by manual reaction times was not affected by target and distractor eccentricity, whereas capture as measured by the eyes was: items close to fixation were more likely to be selected than items presented further away. Furthermore, the effects of target and distractor eccentricity were largely additive, suggesting that the competition between saliency- and relevance-driven selection was modulated by an independent eccentricity-based spatial component. Implications of the dissociation between manual and oculomotor responses are also discussed.

## Introduction

Throughout the day, we look for objects that are relevant to the goals we try to achieve. For example, when we want to watch TV, we might be looking for the remote control on the couch. While doing so, our eyes may inadvertently be drawn to the brightly colored magazine that is also lying on the couch, even though we have no interest at all in reading the magazine at that point in time. This example illustrates that attention and the eyes sometimes select objects that are salient but fully irrelevant in terms of an observer’s goal, a phenomenon denoted as attentional capture.

Attentional capture has been extensively studied using what has become known as the *additional singleton paradigm* (Theeuwes, 1991, 1992). In this paradigm participants are instructed to search for a shape singleton target (e.g., a green diamond) amongst a group of nontargets (e.g., green circles). In the crucial condition, one of the nontargets is replaced by a color singleton distractor (e.g., a red circle). The presence of the distractor typically results in increased reaction times (RTs) compared to trials without a distractor, a finding interpreted as reflecting attentional capture, in that attention is initially shifted to the distractor before it is correctly allocated to the target.

Importantly, under this definition, attentional capture is evoked by a salient stimulus in extrafoveal, eccentric vision. Yet, the effects of eccentricity on attentional capture have rarely been investigated. For instance, to our knowledge, it has never been included as a factor of interest within the additional singleton paradigm, where the target and distractor are typically placed at one and the same eccentricity. This is remarkable because in real life, competing sources of information are usually not present at the same eccentricity, and the competition between relevant and irrelevant salient information (i.e., between a target and distractor) may well be affected by their eccentricities. Neither is eccentricity a factor in currently dominant models of attentional capture, which assume that the activity map representing the relative priority of signals is uniform across the visual field (e.g., Fecteau & Munoz, 2006; Itti & Koch, 2001; Luck et al., 2021; Theeuwes, 2019, for exceptions see: Akbas & Eckstein, 2017; Peters et al., 2005; Zelinsky, 2008, see also Lleras et al., 2022, for a review)

Indeed, it is well known that the visual field is far from homogeneous, as processing changes fundamentally with eccentricity (e.g., Strasburger et al., 2011). This also has effects on selection. For example, it has been shown that RTs increase and processing speed decreases with increasing target eccentricity in simple search tasks (Carrasco et al., 1995; Geweke et al., 2021; Staugaard et al., 2016; Wang et al., 2018; Wolfe et al., 1998; Yeshurun & Carrasco, 1998). This suggests that it takes longer to select more eccentric items, which in turn might also impact attentional capture. Yet target eccentricity has never been systematically manipulated to investigate how it affects capture. This also applies to distractor eccentricity. In one study, Beck and Lavie (2005) showed that a centrally presented distractor caused more interference in a search task than a peripheral one. Using virtually the same task, Chen and colleagues (Chen, 2008; Chen & Treisman, 2008) found the opposite effect, with more interference for peripheral distractors than for central distractors. However, neither of these studies tell us much about attentional capture as such, as distractor interference was manipulated at the level of target-distractor compatibility, while neither saliency nor distractor presence was manipulated. A more informative study in this regard is a study by Wolfe et al (1998), which provides indirect evidence that the relative eccentricity of target and distractors substantially affects visual selection. They asked participants to look for a target that could be presented at one of four levels of eccentricity. The eccentricities of the distractor elements were varied such that in one condition they were presented at the same eccentricity as the target while in another condition they were randomly distributed across the eccentricities so that several distractors were positioned between the target and the central fixation point. The authors found no effect of target eccentricity on search RTs in the equal eccentricity condition, while RTs increased with increasing target eccentricity in the random eccentricity condition. From this the authors derived that in the random condition, attention was first allocated to the intermediate distractors, before the target was selected, suggesting that attentional priority shows eccentricity-dependent biases. In a recent study (van Heusden et al., 2023) we sought to more directly investigate any eccentricity-based attentional biases by presenting observers with displays containing two equally salient and equally relevant targets, which could each be presented at the same or at a different eccentricity. The task was to make an eye movement to either target. We observed a strong bias towards selecting the target closest to fixation, which could not be explained by simple differences in processing speed across eccentricities. Instead, it appeared that attention has a strong preference for objects close to fixation. However, while showing evidence for a bias, this study did not investigate inadvertent capture by salient distractors.

**Fig. 1:**
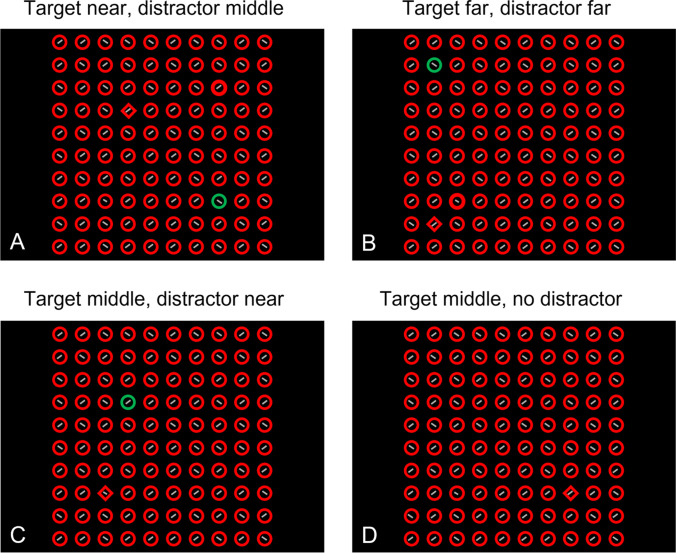
Four examples of the search display. In these examples, the target is the red diamond and the distractor is the green circle. Participants were instructed to find the target and indicate whether the line inside the target was tilted to the left or right from vertical. **A** The target is presented at the near eccentricity and the distractor is presented at the middle eccentricity. **B** Both the target and the distractor are presented at the far eccentricity. **C** The target is presented at the middle eccentricity and the distractor is presented at the near eccentricity. **D** The target is presented at the middle eccentricity and no distractor is present on this trial

Thus, the few studies that have manipulated eccentricity in attention tasks suggest that it can have a considerable effect on prioritization. If so, the eccentricities of target and distractor should also affect attentional capture by salient distractors. Specifically, if attention is center-biased, then we expect salient distractors presented closer to central fixation than the target to lead to more capture than salient distractors that are presented further from central fixation than the target. In the present study we adapted the additional singleton paradigm to systematically investigate the effects of eccentricity on attentional capture and test this specific prediction. Figure 1 illustrates the displays and task. Observers searched for a shape-defined target while ignoring a color-defined distractor, which could be present or absent. Crucially, we independently manipulated target eccentricity as well as distractor eccentricity. Previewing our results, we replicate previous findings showing that the presence of a distractor slows down search (e.g., Theeuwes, 1992). Furthermore, overall RTs increased with target eccentricity, consistent with earlier work (Carrasco et al., 1995; Wolfe et al., 1998). However, and strikingly, neither the eccentricity of the target nor that of the distractor modulated the attentional capture effect as measured by RTs. To further investigate this, in a second experiment we also measured eye movements, and we were able to show that, in contrast to manual RTs, oculomotor capture was affected by both target as well as distractor eccentricity, reflecting a general bias towards items closer by. The findings not only show how attentional capture is shaped by eccentricity, but also how the relatively complex dynamics of the underlying processes can lead to a dissociation between manual and oculomotor indices of attentional capture.

## Experiment 1

### Methods

#### Participants

Due to the COVID pandemic, Experiment 1 was run online. Prior to running the experiment, we ran a power analysis assuming a moderate effect size of 0.5. This showed that a sample size of at least 45 was required to obtain a power of .95 with α = 0.05. As participant exclusion rates can be relatively high for online experiments, we decided to open 70 available timeslots for participation. Ultimately 64 subjects (mean age: 20.9 (SD: 3.0) years; 42 female, 20 male, two other) participated in this experiment. All subjects reported normal or corrected-to-normal vision and gave informed consent prior to participation. Subjects received course credit for their participation. The protocol was approved by the ethics review board of the Faculty of Behavioral and Movement Sciences and the experiment was conducted according to the principles of the Declaration of Helsinki.

#### Stimuli and procedure

The experiment was programmed in JavaScript and run via OSWeb (Mathôt et al., 2012). As this experiment was performed online, without control over viewing circumstances, item sizes are reported in pixels, and color values in RGB. A white (255,255,255) central fixation dot (radius 8 px) was presented on a black (0,0,0) background whenever participants were required to fixate on the center of the display. Four examples of the search display are presented in Fig. 1. Stimuli were circles and diamonds (circle diameter: 26 px, line size diamond: 29 px), presented in either green (0,208,0) or red (255,0,0). The target was defined as one specific combination of shape and color (e.g., a green circle) and remained the same for each participant throughout the entire experiment but was varied (counterbalanced) across participants. The distractor always had a different shape and color than the target. Thus, if the target was a green circle, the distractor was a red diamond. The remaining nontarget elements shared their shape with the distractor, and their color with the target. Stimuli were presented in a 10 x 10 grid of 800 x 800 px, with a center-to-center distance of 78 px in both vertical and horizontal directions. The distractor and target were always presented on the grid’s diagonals – that is, in one of the top-left, top-right, bottom-left, bottom-right quadrants – each at one of three possible eccentricities: 156 px (near), 234 px (middle), and 312 px (far) from the center of the display. Each shape contained a gray (140,139,139) line (width: 3 px, height: 20 px) that was tilted 45° to the left or right from vertical. These lines were intentionally made small, so that participants were not able to perform the orientation judgment from central fixation and instead had to make an eye movement to the target. At the start of the experiment, participants were shown which combination of color and shape was their target. Each trial started with the presentation of a central fixation dot for 500 ms, which was followed by the search display. The search display was presented until keypress. In each trial, participants were instructed to indicate as quickly as possible whether the line inside the target was tilted to the left or to the right, using the z and / keys, respectively.

#### Design

A within-subject design was used with Target Eccentricity (near, middle, far) and Distractor Eccentricity (no distractor, near, middle, far) as factors. There were an equal number of trials for all the different combinations of target and distractor eccentricity, which were presented randomly mixed. Thus, the distractor was present on 75% of the trials (25% for each eccentricity, leaving 25% *no distractor* trials). The combination of color and shape of the target was counterbalanced across participants. Participants completed 50 practice trials and 728 experimental trials, with a break after every 50 trials. During the break, participants were updated on their performance (i.e., the number of errors and average RT). A session took approximately 40 min.

#### Data processing

Prior to running the experiment, we determined that participants would be removed if fewer than 85% of trials were valid. A trial was marked as invalid if: (1) it was not performed at all (i.e., when the experiment was ended prematurely); (2) an invalid response was given (i.e., a key was pressed that was not part of the valid response options); or (3) if the RT on that trial was lower than 200 ms or higher than 4,000 ms. Of the remaining dataset, individual trials on which the RT deviated more than 3 standard deviations from the mean (calculated separately for each unique combination of target and distractor eccentricity) were also discarded.

#### Data analysis

We tested for differences between conditions using a repeated-measures analysis of variance (ANOVA) with α = 0.05. The Greenhouse–Geisser correction was applied if the assumption of sphericity was violated (Greenhouse & Geisser, 1959).

### Results

Based on the exclusion criteria as formulated in the *Methods* section, data of eight participants were discarded as the number of valid trials for these participants fell below 85%, which yielded a final N of 56. For the remaining participants 2.04% of trials exceeded 3 standard deviations from the mean and these trials were also excluded.

#### Task accuracy

Overall mean accuracy was 95.1%. Table 1 shows the mean proportions correct responses separately for the different target and distractor eccentricity conditions. A repeated-measures ANOVA on the individual proportions correct responses with Target Eccentricity (near, middle, far) and Distractor Eccentricity (no distractor, near, middle, far) as factors only revealed a main effect of Target Eccentricity (*F*(1.58, 86.93) = 3.93, *p* < 0.05, η_p_^2^ = 0.07), as accuracy decreased somewhat from near to far eccentricity. There was no effect of Distractor Eccentricity, nor an interaction (*p*s > 0.13). Importantly, at 94% or more, overall accuracy was high. Further analyses focus on RTs.

**Table 1:** Proportions of correct responses (mean and standard deviation) separately for the different target and distractor eccentricity conditions

		Target eccentricity					
		Near		Middle		Far	
		mean	sd	mean	sd	mean	sd
Distractor eccentricity	Near	0.96	0.03	0.95	0.04	0.94	0.06
	Middle	0.96	0.03	0.95	0.03	0.95	0.04
	Far	0.96	0.03	0.95	0.03	0.95	0.05
	No distractor	0.96	0.03	0.95	0.03	0.95	0.05

#### Reaction times

**Fig. 2:**
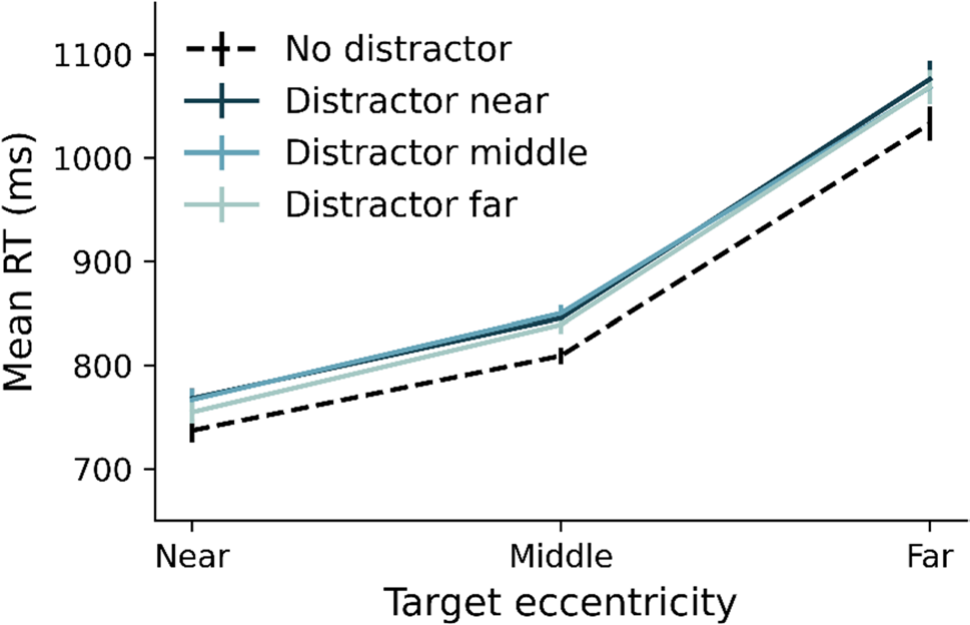
Mean reaction times (RTs) for correct responses as a function of target and distractor eccentricity. Error bars reflect the standard error of the mean across participants

Figure 2 shows the mean RTs for the correct responses only, separately for the different combinations of target and distractor eccentricity including the distractor-absent condition. An omnibus repeated-measures ANOVA on the individual averaged RTs with Target Eccentricity (near, middle, far) and Distractor Eccentricity (no distractor, near, middle, far) as factors revealed a main effect of Target Eccentricity (*F*(1.1, 60.55) = 540.29, *p* < 0.001, η_p_^2^ = 0.91), a main effect of Distractor Eccentricity (*F*(3, 165) = 34.38, *p* < 0.001, η_p_^2^ = 0.38), and no interaction (p > 0.47). As is evident from Fig. 2, RT increased with increasing target eccentricity. Moreover, RTs increased overall with the presence of a distractor. However, beyond the effect of distractor presence, there appeared little effect of actual distractor *eccentricity*. This was confirmed by repeating the ANOVA, but now leaving out the distractor-absent condition. This again revealed a main effect of Target Eccentricity (*F*(1.14, 62.71) = 527.48, *p* < 0.001, η_p_^2^ = 0.91), but now no longer a main effect of Distractor Eccentricity, (*F*(2, 110) = 2.82, *p* = 0.06, η_p_^2^ = 0.05) plus again no interaction (p > 0.58). Thus, the effect of distractor presence remained constant regardless of target eccentricity and distractor eccentricity. It is important to note that with the variation of target and distractor eccentricity, the distance between the two also varied. That is, the distance between target and distractor was on average greater as the singletons were presented at larger eccentricities. It has been shown that target-distractor distance can affect performance in visual search (e.g., Caputo & Guerra, 1998). To examine whether distance played a role in our experiment, we did a control analysis, the results of which are shown in Fig. 6 in the Appendix. This analysis showed that the observed variations in RT could not be explained by differences in target-distractor distance.

### Discussion

Replicating previous findings (e.g., Theeuwes, 1992), the presence of a salient distractor slowed down search, indicating that attention was captured by the salient color singleton. Also replicating previous work (Carrasco et al., 1995; van Heusden et al., 2021; Wolfe et al., 1998), the results show that search times were influenced by target eccentricity, as it takes longer to respond to a target that is presented further away from current fixation. Interestingly, however, the strength of the interference imposed by the distractor was not modulated by the eccentricity of the target and distractor. This is surprising given that objects closer to fixation have been shown to have a competitive advantage over those further into the periphery, resulting in prioritized selection (Staugaard et al., 2016; Wolfe et al., 1998). If objects closer to fixation are more likely to be prioritized for selection, one would have expected stronger attentional capture by distractors that are presented at a location less eccentric than the target. The results suggest this was not the case here, raising questions either about the role of eccentricity in changing the competition between a target and a distractor in visual selection, or about manual response times as a valid measure for mapping out the dynamics of such competition. Before drawing any conclusions on such implications, we decided to repeat the experiment in the laboratory, now using eye tracking in order to obtain a more direct measure of selection behavior.

## Experiment 2

### Methods

#### Participants

Given the better controlled lab environment, we settled for a smaller sample size than in Experiment 1. A planned number of 30 subjects (mean age: 21.3 (SD: 2.5) years; 22 women, eight men), participated. All subjects reported normal or corrected-to-normal vision and gave informed consent prior to participation. Subjects received course credit or money for their participation. The protocol was approved by the ethics review board of the Faculty of Behavioral and Movement Sciences and the experiment was conducted according to the principles of the Declaration of Helsinki.

#### Apparatus

Stimuli were presented on an ASUS ROG Strix monitor with a resolution of 1,920 × 1,080 pixels. Eye movements were recorded using an EyeLink 1000 Plus eyetracker (SR Research, Ontario, Canada). Distance from the screen was kept constant at 70 cm by the use of a chin rest.

#### Stimuli and procedure

Except for some details, stimuli and procedure matched those of Experiment 1. The experiment was programmed in Python and Opensesame (Mathôt et al., 2012). A white (255,255,255) central fixation cross (0.22 dva) was presented on a black (0,0,0) background whenever participants were required to fixate. Stimuli were circles and diamonds (circle diameter: 1.2 dva, line size diamond: 1 dva), presented in either green (6,157,6; 65 cd/m^2^) or red (255,0,0; 64 cd/m^2^). Stimuli were presented in a 10 x 10 grid and were separated by 1.75 dva in both the vertical and the horizontal direction. Distractor and target were presented on the array diagonals, each at one of three possible eccentricities, 4.9 dva (near), 7.4 dva (middle), and 9.9 dva (far) from the center of the display. Each shape stimulus contained a gray (140,139,139) line (width: 0.04 dva, height: 0.27 dva) that was tilted 30° to the left or right from vertical. Before the start of the experiment, a nine-point calibration was performed, and each trial started with a drift-correction.^1^

#### Design

The design also followed Experiment 1. A within-subject design was used with Target Eccentricity (near, middle, far) and Distractor Eccentricity (no distractor, near, middle, far) as factors. All the different combinations of conditions occurred equally often and were presented randomly mixed. The distractor was present on 75% of the trials (25% for each eccentricity, leaving 25% no distractor trials). The combination of color and shape of the target was counterbalanced across participants. Participants completed 48 practice trials and 728 experimental trials, with a break after every 48 trials. During the break, participants were updated on their performance (i.e., the number of mistakes and average RT). A session took approximately 50 min.

#### Data processing and analysis

Eye-movement data were analyzed offline, as follows: Saccade start and end points were defined using the velocity-based algorithm described in Nyström and Holmqvist (2010). Trials on which the starting position of the eye was more than 0.67 dva away from fixation were discarded, as this would hamper the analysis. In order to investigate the extent to which the distractor captured the eyes in the different target and distractor eccentricity conditions, we specified for each trial the sequence of fixations that were sequentially generated. We described these fixation sequences, or scanpaths, as strings of letters, in which a “T” corresponds to a fixation in the target quadrant, “D” corresponds to a fixation in the distractor quadrant, and an “O” corresponds to a fixation in a quadrant other than the target or distractor quadrant. For example, a trial would be labeled “DT” if the eyes first went to the distractor quadrant, and then ended in the target quadrant. We chose quadrant (top-left, top-right, bottom-left, and bottom-right) as the unit of analysis since the target and distractor were always placed on the display diagonals (thus along the center lines of each of the quadrants), and we were interested in any eye movements in the general direction of the distractor or target, rather than exactly landing on them. This is because oculomotor capture is often not complete: eye movements often show undershoots, and such undershoots tend to become larger with eccentricity (which would demand flexible criteria for what counts as an “on-item” fixation; Dick et al., 2004; van Opstal & van Gisbergen, 1989). Furthermore, by adopting a broad criterion for the inclusion of eye movements, we could rely on virtually all trials, which was important for our comparison of the manual RT patterns across different eye-movement trajectories. As we were interested in target and distractor selection only, we further simplified the scanpath analyses by absorbing visits to quadrants that contained neither the target nor the distractor into the main sequences. For example, ODT and DOT were both classified as DT. If the eyes never visited the target or distractor quadrant, it received the letter “O” for *other*. In addition, multiple consecutive fixations within one quadrant were grouped together (i.e., TDDT becomes TDT). Next, for each scanpath separately we calculated the arrival time in the first quadrant (either target or distractor depending on the scanpath) and the total dwell time within a quadrant (aggregated across multiple consecutive fixations in that quadrant when applicable). Time spent in one of the other quadrants did not contribute to dwell time.

### Results

Trials in which RT was higher than 4,000 ms or was lower than 200 ms (0.16%) were excluded from the analyses. Of the remaining trials, a further 14% were discarded because the first eye movement started more than 0.67 dva away from fixation.

#### Task accuracy

Overall mean accuracy was 93.1%. Table 2 shows the mean proportions of correct responses separately for the different target and distractor eccentricity conditions. A repeated-measures ANOVA on the individual proportions correct responses with Target Eccentricity (near, middle, far), and Distractor Eccentricity (no distractor, near, middle, far) as factors revealed a main effect of Target Eccentricity (*F*(1.17, 34.07) = 6.83, *p* < 0.01, η_p_^2^ = 0.19). As in Experiment 1, accuracy was overall high, but decreased with target eccentricity. There was no effect of Distractor Eccentricity nor an interaction (*p*s > 0.62). Again, we focus on the RTs.

**Table 2:** Proportions of correct responses (mean and standard deviations) separately for the different target and distractor eccentricity conditions

		Target eccentricity					
		Near		Middle		Far	
		mean	sd	mean	sd	mean	sd
Distractor eccentricity	Near	0.94	0.07	0.93	0.08	0.91	0.10
	Middle	0.94	0.06	0.93	0.07	0.91	0.10
	Far	0.94	0.05	0.93	0.07	0.92	0.10
	No distractor	0.95	0.06	0.93	0.08	0.92	0.09

#### Reaction times

**Fig. 3:**
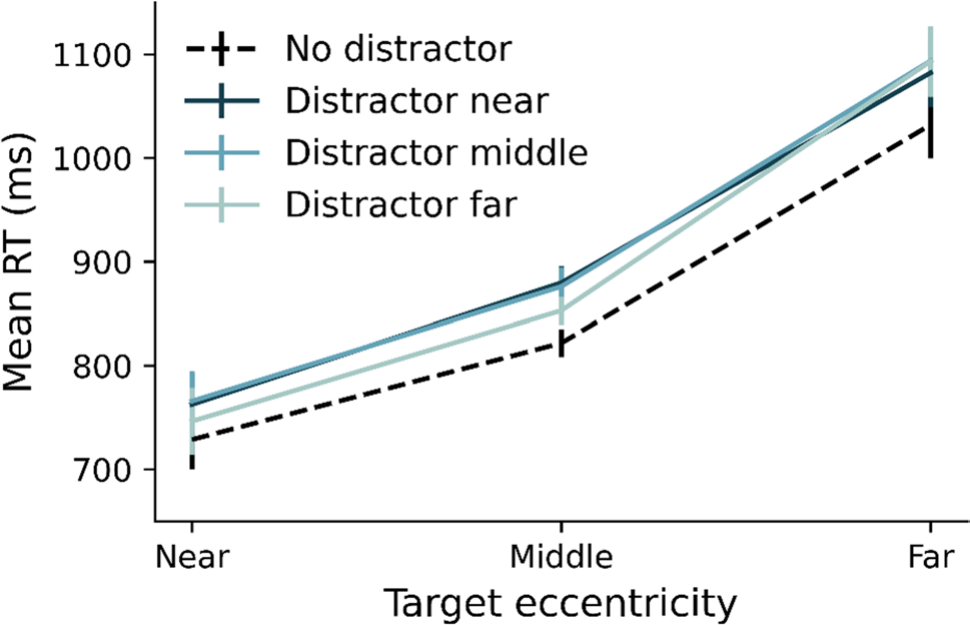
Mean reaction times (RTs) in milliseconds as a function of target and distractor eccentricity. Error bars reflect standard error across participants

Figure 3 shows the mean RTs for correct responses only, for all possible target and distractor eccentricities. The pattern confirms that of Experiment 1. A repeated-measures ANOVA on the individual averaged RTs with Target Eccentricity (near, middle, far), and Distractor Eccentricity (no distractor, near, middle, far) as factors revealed a main effect of Target Eccentricity (*F*(1.24, 35.95) = 106.21, *p* < 0.001, η_p_^2^ = 0.79), a main effect of Distractor Eccentricity (*F*(3, 87) = 22.07, *p* < 0.001, η_p_^2^ = 0.43), and no interaction (*p* = 0.08). As is evident from Fig. 3, RT increased with increasing target eccentricity. Moreover, RTs increased overall with the presence of a distractor. However, beyond the effect of distractor presence, there appeared little effect of actual distractor eccentricity. This was confirmed by repeating the ANOVA without the distractor-absent condition. This again revealed a main effect of Target Eccentricity (*F*(1.3, 37.65) = 104.19, *p* < 0.001, η_p_^2^ = 0.78), but now no longer a main effect of distractor eccentricity, (*F*(2, 58) = 2.32, *p* = 0.11, η_p_^2^ = 0.07) plus again no interaction (p > 0.17). Again, the absolute distance between target and distractor location could not account for the observed variations in RT (see Fig. 6). In sum, as in Experiment 1, the effect of distractor presence remained largely constant across target and distractor eccentricities. Thus, the RT pattern again suggests that attentional capture is not modulated by the eccentricity of target and distractor.

#### Eye movements – proportions to target and distractor

To analyze the eye movements, we first mapped out the different scanpaths that were followed when observers searched for the target (see *Methods* for further details). We observed 12 different scanpaths and Table 3 shows the proportions of trials on which each of these occurred. Notably, 95.1% of all fixation sequences followed either the T-scanpath (i.e., preferring the target quadrant over the distractor quadrant; 56.5%) or the DT-scanpath category (i.e., preferring the distractor quadrant before moving towards the target quadrant; 38.6%). For the sake of simplicity, further analyses were limited to these two categories only. The proportions of trials in which the eyes followed either one of these two scanpaths provides an index of the extent to which the initial eye movements were directed toward the target or the distractor.

**Table 3:** Results of the scanpath analysis

Scanpath	Percentage	Scanpath	Percentage
T	56.5%	DT	38.6%
TD	0.01%	DTD	1.92%
TDT	0.67%	DTDT	0.33%
TDTD	0.05%	DTDTD	0.03%
TDTDT	0.04%	DTDTDT	0.01%
D	0.38%	O	0.37%

*T* target, *D* distractor, *O* other

**Fig. 4:**
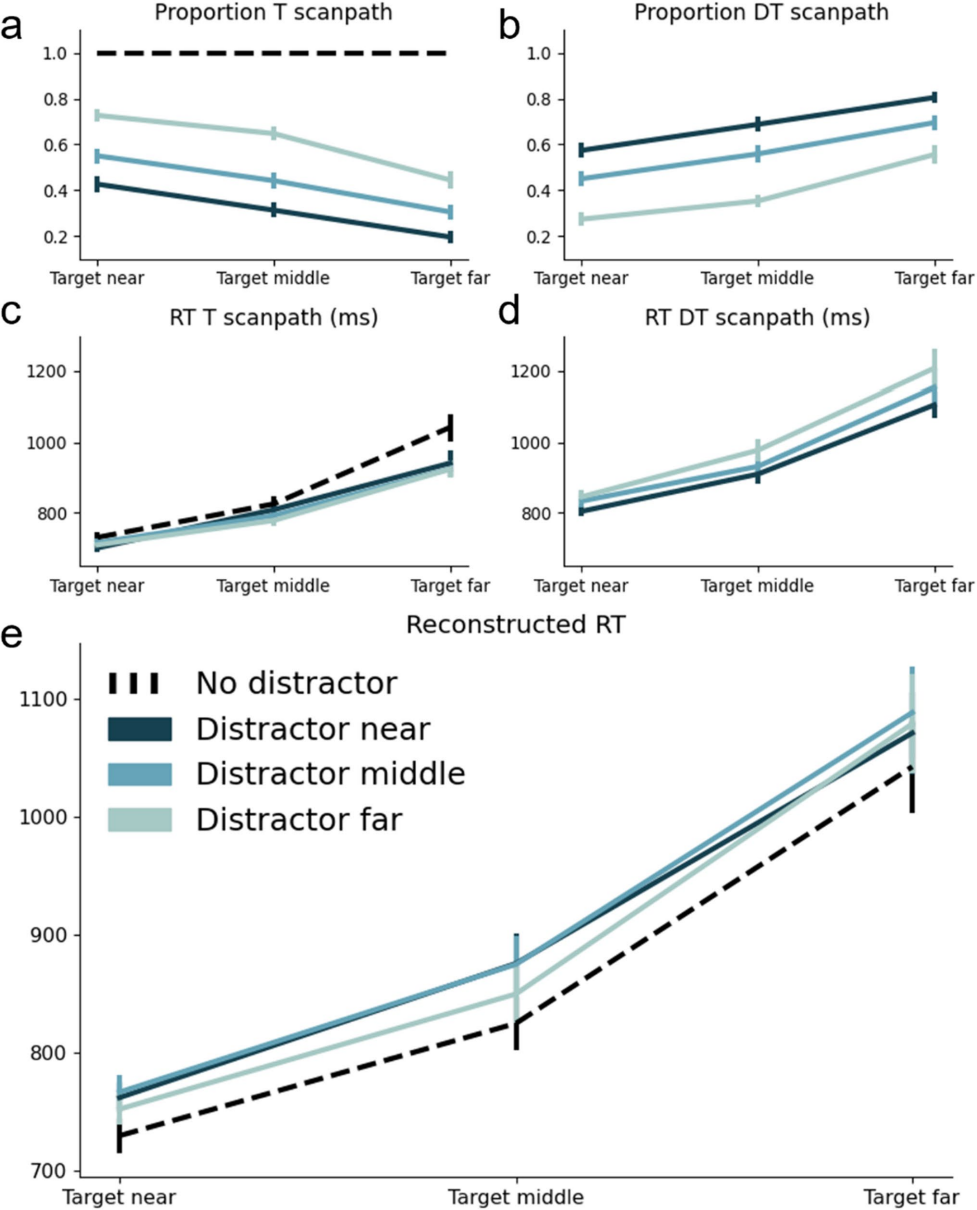
Average proportions of following the T (target) (**a**) or DT (distractor-target) (**b**) scanpath plotted separately for all unique combinations of target and distractor eccentricity. Note that p(DT) = 1 – p(T). Average reaction time (RT) in milliseconds for the T scanpath (**c**) and the DT scanpath (**d**) plotted separately for all unique combinations for target and distractor eccentricity. **e** Reconstructed RT that was calculated by: (p(T) x RT T) + (p(DT) x RT DT). Error bars reflect standard errors across participants

As a next step we computed the probability of observers following the T or DT scanpath in dependency of target and distractor eccentricity, as shown in Fig. 4a and b. This immediately shows a pattern quite different from the manual RTs, as the likelihood of following a particular scanpath was affected by the eccentricity of not only the target, but also the distractor singleton. Specifically, the eyes were less likely to go straight to the target (and more likely to first go to the distractor) when the target was further away from the center, and when the distractor was closer to the center. The statistical analyses confirmed this pattern: A repeated-measures ANOVA with the proportions of following the T scanpath as dependent measure, and with Target Eccentricity (near, middle, far) and Distractor Eccentricity (no distractor, near, middle, far) as factors revealed a main effect of Target Eccentricity (*F*(1.46, 42.45) = 132.91, *p* < 0.001, η_p_^2^ = 0.82; with all pairwise comparisons being significant (all *F*-values > 79.67, all* p*-values < 0.001), a main effect of Distractor Eccentricity (*F*(1.98, 57.43) = 296.35, *p* < 0.001, η_p_^2^ = 0.91; with again all pairwise comparisons being significant (all *F*-values > 84.15, all* p*-values < 0.001). The interaction between Target Eccentricity and Distractor Eccentricity was also significant (*F*(3.57,103.41) = 23.88, *p* < 0.001, η_p_^2^ = 0.45), but was primarily driven by distractor presence: Without the no distractor condition, the interaction was no longer significant (*F*(2.83,82.06) = 2.21, *p* = 0.1, η_p_^2^ = 0.07). While we cannot exclude a weak interaction effect with more power, we believe it is safe to say that the effects of target eccentricity and distractor eccentricity were largely additive. In Fig. 4 we can see that whenever the target and distractor are presented at the same eccentricity, observers are almost equally likely to select either one of the items. Whenever the target and distractor are presented at different eccentricities, the one closer to fixation is more likely to be selected. In other words, the initial eye movements are more likely to be directed to the distractor quadrant when target eccentricity increases and when distractor eccentricity decreases. Also here, we found that the absolute distance between target and distractor location could not account for these results (see Fig. 6).

How then can this pattern be reconciled with the overall pattern of manual RTs, where target and distractor eccentricity did not alter the interference caused by the presence of the distractor effect? For this it is instructive to look at the manual RTs for each of the two scanpaths, as are shown in Fig. 4c and d. We report the full statistics with and without the no-distractor condition in Table 4,^2^ in the Appendix**.** Here we summarize the main findings: RT increased with target eccentricity, but this increase was stronger for DT scanpaths (Fig. 4d) than T scanpaths (Fig. 4c), as there was a significant Scanpath X Target Eccentricity interaction effect (*F*(1.2,33,64) = 10.05, *p* < 0.001, η_p_^2^ = 0.26). Moreover, for the T scanpaths, the increase in RT as a function of target eccentricity is larger in the no-distractor condition than the other conditions, as the interaction between target and distractor eccentricity is significant when the no-distractor condition is included (*F*(2.76,79.98) = 3.78, *p* < 0.05, η_p_^2^ = 0.12) but not when it is excluded (*F*(2.14,62.17) = 0.66, *p* = 0.66, η_p_^2^ = 0.02). The absence of any interaction between target eccentricity and distractor presence such as in the overall RT pattern (Fig. 3) can then be explained by combining these differential RT patterns depicted in Fig. 4c and 4d with the differential proportions depicted in Fig. 4a and b – as becomes evident from the reconstruction of the weighted average RT pattern which is shown in Fig. 4e.^3^ The same goes for the effect of distractor eccentricity. RT increases with distractor eccentricity but only for the DT scanpaths (Fig. 4d) and not for the T-scanpaths (Fig. 4c). Combined, the differential RT pattern for the different scanpaths (Fig. 4c and d) and the differential proportions for these scanpaths (Fig. 4a and b), explain the null effect of distractor eccentricity in the overall RT pattern (Fig. 3): while distractors far away led to longer delays when they captured the eyes, they were less likely to capture the eyes in the first place, thus allowing for a larger proportion of relatively fast target-only trajectories.

#### Eye movements – latency effects

**Fig. 5:**
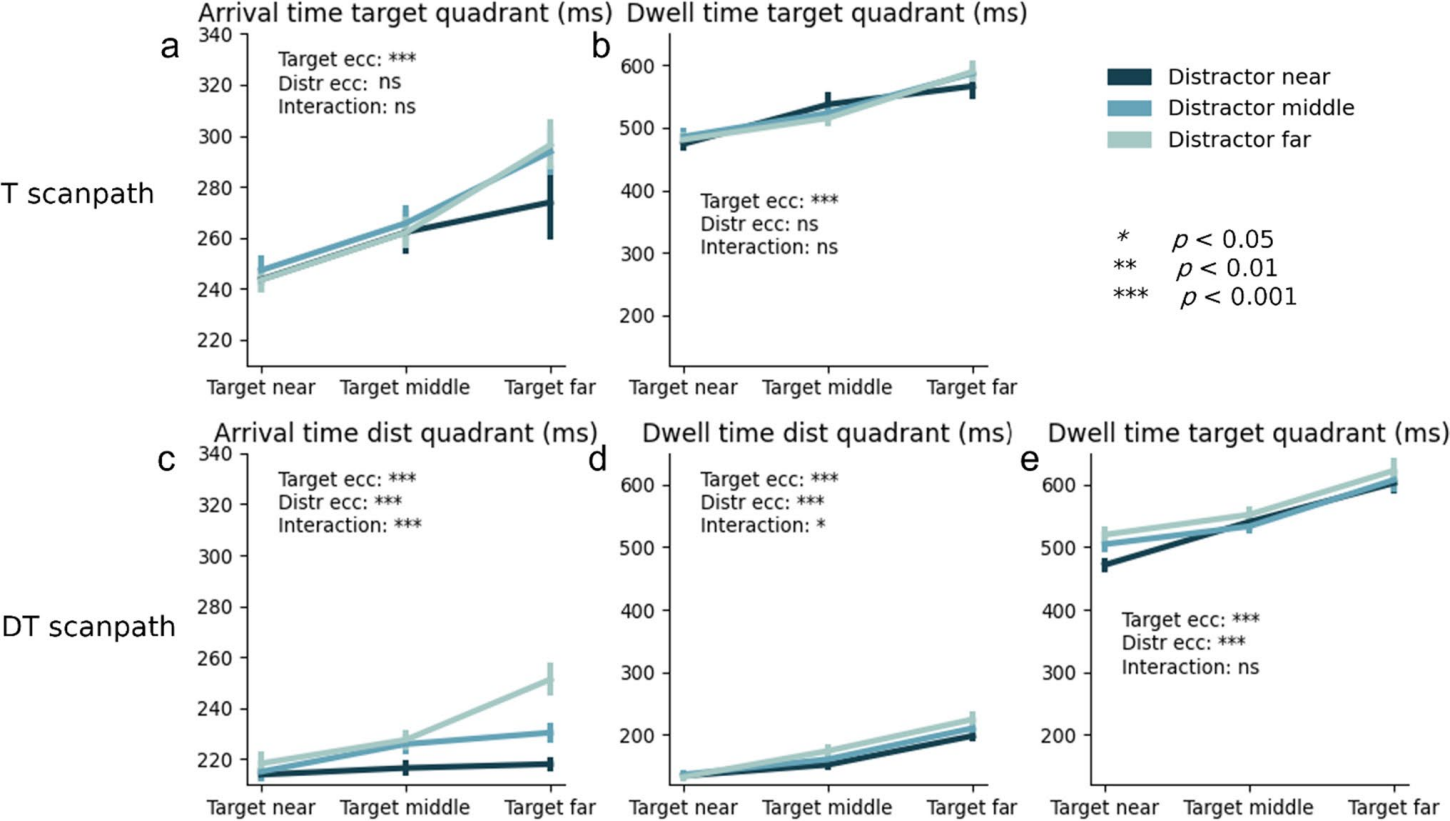
T (target) scanpath: Arrival time in the target quadrant (**a**) and dwell time in the target quadrant (**b**). DT (distractor-target) scanpath: Arrival time in the distractor quadrant (**c**), dwell time in the distractor quadrant (**d**) and dwell time in the target quadrant (**e**). Error bars reflect standard error across participants

**Fig. 6:**
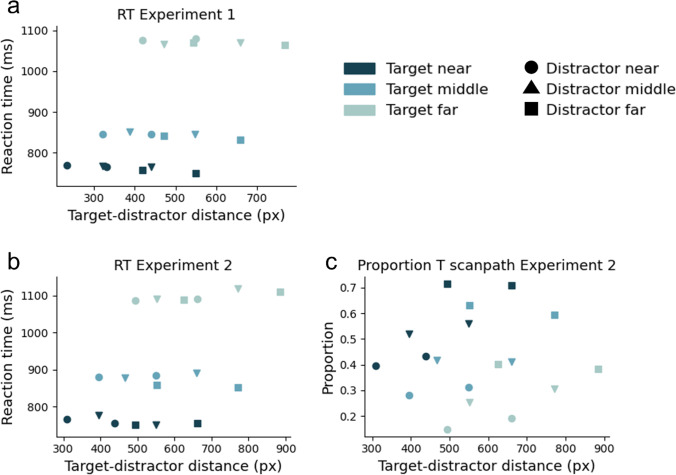
Performance as a function of target-distractor distance. The markers indicate at which target eccentricity (colors) and distractor eccentricity (shapes) the elements were presented. **a** Average manual reaction time in Experiment 1 plotted as a function of target-distractor distance. Overall, there is a positive correlation between target-distractor distance and reaction time (RT) (r = 0.53, *p* = 0.02). However, when looking at the figure, we can see that data points cluster together based on target eccentricity. When we control for target eccentricity, we find the following partial correlation between target-distractor distance and RT: r = -0.11, *p* = 0.67. This shows that the positive correlation between target-distractor distance and RT is entirely due to variations in target eccentricity and not to variations in target-distractor distance. **b** Average manual RT in Experiment 2 plotted as a function of target-distractor distance. The overall correlation between target-distractor distance and RT is 0.53 (*p* = 0.03). Again, data points cluster together based on target eccentricity. When we control for target eccentricity, we find the following partial correlation between target-distractor distance and RT: r = 0.005, *p* = 0.98. **c** Average proportion of trials in which participants followed the T scanpath in Experiment 2 as a function of target-distractor distance. There is no significant correlation between target-distractor distance and proportion T scanpath (r = 0.03, *p* = 0.88), indicating that target-distractor distance does not explain the observed variations in selection behavior. Instead, data points again cluster together based on shading, representing a main effect of target eccentricity. We also observe a clustering on the basis of symbols, which reflects the effect of distractor eccentricity

To investigate how the RT differences between T and DT scanpaths were generated, we further decomposed the scanpaths into arrival time in the first quadrant (either target or distractor depending on the scanpath) and the total dwell time within each quadrant (see Fig. 5). We summarize the most important statistics here, while all individual *F, p* and η_p_^2^ statistics can be found in Table 4 in the Appendix (including comparisons to the no distractor condition, which are left out here). First, Fig. 5a and b show the average arrival time, and the average subsequent dwell time within the target quadrant for the T scanpath. Both measures show a main effect of Target Eccentricity (all *F*-values > 27.95, all* p*-values < 0.001), as with increasing target eccentricity the average arrival time and dwell time within the target quadrant increases. None of the measures were affected by distractor eccentricity (whether main effect or interaction).

Figure 5c, d, and e show average arrival time within the distractor quadrant, dwell time in the distractor quadrant and dwell time in the target quadrant for the DT scanpath. When comparing the T scanpath to the DT scanpath, two things stand out. First, arrival time in the distractor quadrant is lower than arrival time in the target quadrant. This is in line with previous findings showing that saliency-driven eye movements (i.e., to a salient distractor) occur quickly, whereas relevance-driven eye movements (i.e., to a relevant target) take longer to initiate (e.g., van Heusden et al., 2022; Van Zoest et al., 2004). Second, dwell times were overall lower in distractor than in target quadrants. This makes sense, as participants need to discriminate and respond to the orientated line segment inside the target, neither of which is the case for the distractor.

Also for the DT scanpath, all measures again showed a main effect of Target Eccentricity (all *F*-values > 25.56, all* p*-values < 0.001). With increasing target eccentricity, the average arrival time in the distractor quadrant increased, as well as the average dwell time within the distractor quadrant and target quadrant. Moreover, all measures also showed a main effect of Distractor Eccentricity (all *F*-values > 7.87, all* p*-values < 0.001). With increasing distractor eccentricity both the average arrival time in the distractor quadrant and the average dwell time within the distractor quadrant and target quadrant increased. The results also reveal Target Eccentricity x Distractor Eccentricity interactions for both the arrival time and the dwell time in the distractor quadrant (both *F-*values* >* 3.15, both *p*-values < 0.05): The latency advantage for close-by distractors was particularly large when the target itself was far away. However, notice that these initial latency interactions may have been offset by the numerically larger, yet non-reliable *opposite* interaction pattern for the dwell time in the target quadrant, thus eventually contributing to the *additivity* of effects on the eventual manual RT.

### Discussion

Taking overall manual RT as the dependent measure, we again found a strong interference effect of the distractor as manual RT was generally larger in the presence than in the absence of a distractor, consistent with the distractor capturing attention. Importantly, this effect was not modulated by the eccentricities of the target or distractor. There was again an overall main effect of target eccentricity, but target eccentricity did not interact with distractor presence, and there was little to no effect of distractor eccentricity. These results mimic those of Experiment 1, and would, taken at face value, suggest again that attentional capture is insensitive to variations in the eccentricity of target and distractor.

However, the eye-movement data suggest a rather different story. Most notably, the proportion of oculomotor capture by the distractor was profoundly affected by the eccentricities of both the target and the distractor. The closer a target or a distractor is to central fixation, the more likely the initial eye movement will be directed to that singleton, consistent with the idea that items closer to fixation are generally prioritized over those further away. This pattern can be reconciled with the overall pattern of RTs if we consider not only selection behavior itself but also the RTs obtained for the different scanpaths. The effect of target eccentricity on RT is larger for DT scanpaths than for T scanpaths. Yet, if the eyes happen to correctly select the target in the presence of a salient distractor, the effect of target eccentricity is actually even smaller than when no distractor is presented. It appears that by reflecting only the end result of the process, the manual RTs obscure these underlying dynamics, thus falsely suggesting that capture remains constant with target eccentricity. The same applies to the effect of distractor eccentricity. More eccentric distractors are less likely to capture the eyes than less eccentric distractors, but when they do, they lead to additional slowing whereas this is not the case when the eyes immediately select the target. Combining the relative proportions and response times then leads to a null effect of distractor eccentricity on attentional capture.

Finally, a more detailed analyses of eye movement latencies revealed a number of subtle interactions in terms of orienting towards (as indexed by arrival time in the target or distractor quadrant) a target or distractor and dwelling on the object before moving away (in case of a distractor) or making a decision (in case of a target). These latencies again suggested an advantage for distractors closer to fixation, as they resulted in shorter orienting latencies, especially when the target was further away.

## General discussion

Attentional capture by salient stimuli is a ubiquitous phenomenon that has been extensively studied. While attentional capture is driven by eccentric vision, little is actually known about whether and how capture is modulated by eccentricity. In the current study, we used the additional singleton paradigm in two experiments to systematically investigate the effects of target and distractor eccentricity on attentional capture.

We found that the presence of an irrelevant color singleton slowed down search times, consistent with the idea that it captured attention. At the same time, the manual RT results suggested that the strength of this attentional capture effect was not modulated by item eccentricity. At face value this finding runs counter to earlier indications that attention is biased towards more central stimuli (Van Heusden et al., 2023; Wolfe et al., 1998; see also Staugaard et al., 2016). If so, then distractors should have interfered more when presented more centrally than the target. Given that this was not the case, we would have to conclude that either the strength of attentional capture is homogeneous across the visual field, or manual RTs do not provide a correct indication of attentional capture in this respect. The eye-movement behavior observed in Experiment 2 strongly suggests the latter. We found that the likelihood of orienting towards a distractor clearly depended on both the target eccentricity and the distractor eccentricity. In contrast to the RTs, this indicates that capture is profoundly affected by target and distractor eccentricity. Interestingly, target and distractor eccentricity influenced oculomotor capture in a largely additive way, suggesting that target and distractor eccentricity do not simultaneously act on the same process.

### Dissociation between eye movements and manual reaction times

The finding that attentional capture was modulated by target and distractor eccentricity in eye movements but not in manual RTs can be understood by looking at the search times separately for different scanpaths. Specifically, while distractors far away led to longer delays *when* they captured the eyes, they were less likely to capture the eyes in the first place, thus allowing for a larger proportion of relatively fast target-only trajectories. The important conclusion to be drawn from this is that one should not take manual RTs at face value as providing an index of attentional capture. The RT difference between distractor present and distractor-absent conditions has been a hallmark of attentional capture since the introduction of the additional singleton paradigm (Theeuwes, 1992; see also Luck et al., 2021) and is probably still the most frequently used dependent measure. While we do not wish to argue that the distractor presence effect on RT does not at all reflect attentional capture ( for that argument, see, e.g., Folk & Remington, 1998; Wykowska & Schubö, 2011), the current findings suggest that a presence or absence of any modulation of such effects need not necessarily have a relation to underlying attentional capture effects as revealed by eye movements. Of course, this line of reasoning relies on the premise that eye-movement measures (here specifically the proportion orienting towards the distractor) provide a more veridical index of attentional capture than manual RTs. We believe there is sufficient reason to assume this is the case. For one, in terms of latency, eye movements occur earlier and therefore closer to the sensory processes of interest than manual RTs, which reflect a chain of additional decision and response selection processes. Second, in contrast to manual RTs, eye movements show a clear directionality towards the object of interest, commensurate with the spatial selection process assumed to underlie attentional capture. Last but not least, directing attention has been shown to be a prerequisite for directing the eyes (Deubel & Schneider, 1996; Hoffman & Subramaniam, 1995). In conclusion, as we have shown here, manual RTs may obscure the more complex underlying dynamics of attentional capture, and oculomotor measure should therefore be the preferred measure of choice – certainly when studying attentional priorities across eccentricity.

### Oculomotor capture in previous studies

Previous studies using the additional singleton paradigm have also shown that the eyes are captured by an irrelevant salient singleton (e.g., Adams & Gaspelin, 2020; Gaspelin et al., 2017; Gaspelin & Luck, 2019; Mulckhuyse et al., 2008; Theeuwes et al., 1998, 1999, 2003; Wu & Remington, 2003). However, in many of these studies, observers searched for a shape singleton in the presence of an irrelevant color singleton without knowing the precise target and distractor features, which may have precluded strong top-down, relevance-driven guidance of attention (e.g., Adams & Gaspelin, 2020; Gaspelin et al., 2017, 2019; Theeuwes et al., 2003; Wu & Remington, 2003). In contrast, studies in which observers knew the exact target shape, as in our present study, capture of the eyes by a color distractor was found to be negligible, while still observing costs in terms of manual RTs (Theeuwes, et al., 2003; Wu & Remington, 2003). How can the clear oculomotor capture in our Experiment 2 be reconciled with the lack of such capture in these previous studies? One potential factor here is the display arrangement. While previous studies using this type of task have typically used sparse displays with fewer than ten items arranged at equal distance around the center, we used much denser displays consisting of many more items. It has been shown that denser displays increase the saliency of a singleton (e.g., Bravo & Nakayama, 1992; Meeter & Olivers, 2006; Theeuwes, 2004). Indeed, studies using denser displays have consistently demonstrated that irrelevant color singletons capture the eyes (Heimler et al., 2014, 2015; van Heusden et al., 2022; van Zoest & Donk, 2005; Van Zoest & Donk, 2004, 2008).

### Target and distractor eccentricity effects in eye movements and implications for the priority map

Models of attentional control commonly assume selection to be guided by the activity distribution in a priority map (Fecteau & Munoz, 2006; Itti & Koch, 2001; Luck et al., 2021; Theeuwes, 2019), which is a spatial representation ranking individual locations in the visual field in order of their relative priority for selection. The priority values in the map can be shaped by various influences among which not only saliency and relevance are prominent, but also contextual factors, such as previously rewarding experiences and statistical regularities (for other influences, see Awh et al., 2012; Luck et al., 2021; Wolfe & Horowitz, 2017). The fact that there is more capture by the irrelevant color singleton when target eccentricity increases and distractor eccentricity decreases indicates that the competition between target and distractor is further shaped by their respective eccentricities, such that items closer to the central fixation point are given more priority than those further away. An important question then is how such eccentricity-dependent changes in priority are produced.

Our data cannot provide a conclusive answer to this, but one way in which this could be achieved is by spatially weighing the signals in the priority map (see, e.g., Luck et al., 2021). It is commonly assumed that the priority of specific locations can be selectively increased or decreased through spatial weight gain control, for instance as induced by spatial cueing (Müller & Rabbitt, 1989; Posner, 1980), or statistical learning (Ferrante et al., 2018; Huang et al., 2021, 2022; Luck et al., 2021; Wang & Theeuwes, 2018). Possibly eccentricity acts in a similar manner, in that it operates on the values in the priority map through spatial gain control, such that less eccentric items receive stronger weights than those presented further away. This idea bears much resemblance to the central bias notion as proposed by Wolfe et al. (1998) to account for the eccentricity effects in their study (see also Feng & Spence, 2017) and is also consistent with our finding that both target and distractor eccentricity affect capture. However, our results also showed that the effects of target eccentricity and distractor eccentricity were largely additive – in other words, the eccentricity of one item did not modulate the effect of the eccentricity of the other item. This suggests that the two objects were not in direct competition with each other, which goes against this idea.

One way to explain this is to assume a role for *time*. Possibly, eccentricity modulates the speed at which the priority map receives input from early visual processes, such that activity accumulates faster at less eccentric locations than at more eccentric locations (though see Carrasco et al., 2003; Jovanovic & Mamassian, 2020; McKee & Taylor, 1984). As a result, the location of a less eccentric item becomes active at an earlier point in time than that of a more eccentric item, which in turn biases selection towards the former. Importantly, according to this account, changing an item’s eccentricity does not necessarily lead to a modulation of the *strength* of activity at its corresponding location in the priority map as such, but rather to a shift in the moment at which the activity emerges. Consequently, an item’s location may temporarily be prioritized over another item that emerges later, independently of the other factors affecting priority. Current conceptions of the priority map typically do not include time as a crucial factor, for selection priority is commonly conceptualized in terms of a two-dimensional spatial map ranking individual locations solely in terms of activity strength rather than when they become activated. The present account is based on the idea that this map is essentially three-dimensional in that it does not only outline how active different locations are but also when activity arises. By incorporating time as a third dimension, the priority map is conceptualized as a continuously changing landscape that dynamically shapes selection behavior (Donk, 2021; Donk & van Zoest, 2011; Donk & Van Zoest, 2008; van Heusden et al., 2021; Van Zoest & Donk, 2008). The present results are in line with this notion and suggest that eccentricity further modulates the timing of events in the priority map. Note further that under this notion we do not consider a purely low-level visual (Carrasco et al., 1995) and an attentional account (Wolfe et al., 1998) to be mutually exclusive but rather complementary. Eccentricity affects low-level vision that may involve changes in the contrast computations that provide the saliency signal, as well as the signal for any top-down initiated modulations to work with, thus changing the dynamic landscape of the priority map to the benefit of more central vision. In fact, the inherent advantage of central vision may well result in a self-reinforcing mechanism such that due to visibility factors, selection of near-by information is more likely to be successful and rewarding (i.e., the desired object is being selected) than selection of information further away. A lifelong experience with nearby information being more reliable than information further in the periphery is likely to result in a consistent and universal attentional bias in favor of the first.

## Conclusion

In summary, our findings show that item eccentricity modulates attentional capture: items presented close to fixation are more likely to be selected than items presented further away. Given the nature of the eccentricity-based modulations we propose a general but dynamic spatial bias towards more central stimuli which may consecutively affect saliency and relevance signals. In any case, eccentricity is an important factor that strongly determines selection and that should therefore deserve a firm place in models of attention.

## Figures and Tables

**Table 4:**
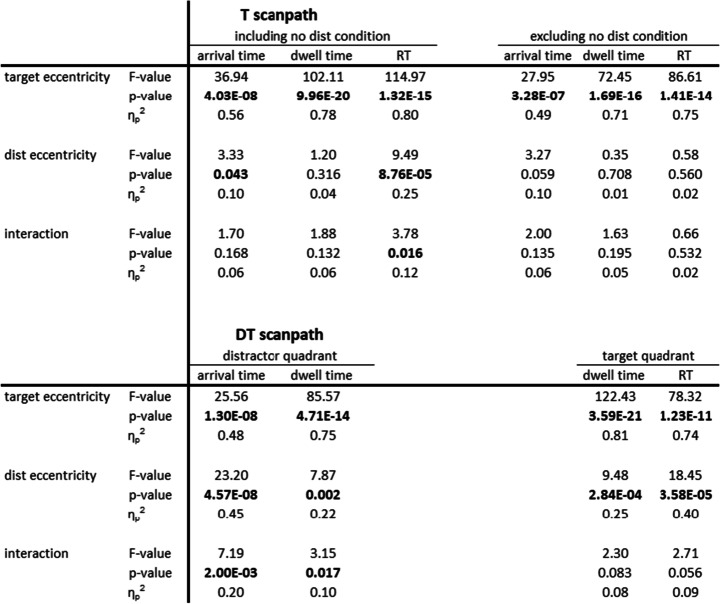
Statistics describing arrival time, dwell time and reaction times of the T and DT scanpath

For the T scanpath the same analysis was repeated twice, once including the no distractor condition (left) and once excluding the no distractor condition (right)

## Data Availability

All of the data and experiments are available via the Open Science Framework and can be accessed online: https://osf.io/gsaue/

## Footnotes

1 Due to a programming error the orientation of the gray line inside the target shape was consistently opposite to that of the gray line in the distractor shape for the first seven participants in the experiment. This error was corrected for the remaining participants. Later analyses showed that there was no difference in the pattern of results between the first seven participants and the other participants, suggesting that this error was not critical.

2 One participant never followed the DT scanpath when the target was near and the distractor was far, and thus could not be entered in the ANOVAs describing the DT scanpath.

3 Note that these computations and therefore Fig. 4e are based on slightly fewer trials (i.e., 95.1%) than Fig. 3, given the sub-selection of scanpaths. A weighted average was calculated for each combination of target and distractor eccentricity separately.

## Code availability

All of the experimental analyses are available via the Open Science Framework and can be accessed online: https://osf.io/gsaue/

## References

[CR1] Adams OJ, Gaspelin N (2020). Assessing introspective awareness of attention capture. Attention, Perception, and Psychophysics..

[CR2] Akbas E, Eckstein MP (2017). Object detection through search with a foveated visual system. PLoS Computational Biology.

[CR3] Awh E, Belopolsky AV, Theeuwes J (2012). Top-down versus bottom-up attentional control: A failed theoretical dichotomy. Trends in Cognitive Sciences.

[CR4] Beck DM, Lavie N (2005). Look Here but Ignore What You See: Effects of Distractors at Fixation. Journal of Experimental Psychology: Human Perception and Performance.

[CR5] Bravo MJ, Nakayama K (1992). The role of attention in different visual-search tasks. Perception & Psychophysics.

[CR6] Caputo G, Guerra S (1998). Attentional selection by distracter suppression. Vision Research.

[CR7] Carrasco M, Evert DL, Chang I, Katz SM (1995). The eccentricity effect: Target eccentricity affects performance on conjunction searches. Perception & Psychophysics.

[CR8] Carrasco M, McElree B, Denisova K, Giordano AM (2003). Speed of visual processing increases with eccentricity. Nature Neuroscience.

[CR9] Chen Z (2008). Distractor eccentricity and its effect on selective attention. Experimental Psychology.

[CR10] Chen Z, Treisman A (2008). Distractor inhibition is more effective at a central than at a peripheral location. Perception and Psychophysics.

[CR11] Deubel H, Schneider WX (1996). Saccade target selection and object recognition: Evidence for a common attentional mechanism. Vision Research.

[CR12] Dick S, Ostendorf F, Kraft A, Ploner CJ (2004). Saccades to spatially extended targets: The role of eccentricity. NeuroReport.

[CR13] Donk M (2021). The progress revisited: How the dispute between stimulus-driven and contingent-capture advocates is hampered by a blindness for change. Visual Cognition.

[CR14] Donk M, Van Zoest W (2008). Effects of salience are short-lived. Psychological Science.

[CR15] Donk M, van Zoest W (2011). No control in orientation search: The effects of instruction on oculomotor selection in visual search. Vision Research.

[CR16] Fecteau JH, Munoz DP (2006). Salience, relevance, and firing: A priority map for target selection. Trends in Cognitive Sciences.

[CR17] Feng J, Spence I (2017). The effects of spatial endogenous pre-cueing across eccentricities. Frontiers in Psychology.

[CR18] Ferrante O, Patacca A, Di Caro V, Della Libera C, Santandrea E, Chelazzi L (2018). Altering spatial priority maps via statistical learning of target selection and distractor filtering. Cortex.

[CR19] Folk CL, Remington R (1998). Selectivity in distraction by irrelevant featural singletons: Evidence for two forms of attentional capture. Journal of Experimental Psychology: Human Perception and Performance..

[CR20] Gaspelin N, Luck SJ (2019). Inhibition as a potential resolution to the attentional capture debate. Current Opinion in Psychology.

[CR21] Gaspelin N, Leonard CJ, Luck SJ (2017). Suppression of overt attentional capture by salient-but-irrelevant color singletons. Attention, Perception, and Psychophysics.

[CR22] Gaspelin N, Gaspar JM, Luck SJ (2019). Oculomotor inhibition of salient distractors: Voluntary inhibition cannot override selection history. Visual Cognition.

[CR23] Geweke F, Pokta E, Störmer VS (2021). Spatial distance of target locations affects the time course of both endogenous and exogenous attentional deployment. Journal of Experimental Psychology: Human Perception and Performance.

[CR24] Greenhouse SW, Geisser S (1959). On methods in the analysis of profile data. Psychometrika.

[CR25] Heimler B, Pavani F, Donk M, van Zoest W (2014). Stimulus- and goal-driven control of eye movements: Action videogame players are faster but not better. Attention, Perception, and Psychophysics..

[CR26] Heimler B, van Zoest W, Baruffaldi F, Donk M, Rinaldi P, Caselli MC, Pavani F (2015). Finding the balance between capture and control: Oculomotor selection in early deaf adults. Brain and Cognition.

[CR27] Hoffman JE, Subramaniam B (1995). The role of visual attention in saccadic eye movements. Perception & Psychophysics.

[CR28] Huang C, Vilotijević A, Theeuwes J, Donk M (2021). Proactive distractor suppression elicited by statistical regularities in visual search. Psychonomic Bulletin and Review.

[CR29] Huang C, Donk M, Theeuwes J (2022). Proactive enhancement and suppression elicited by statistical regularities in visual search. Journal of Experimental Psychology: Human Perception and Performance.

[CR30] Itti L, Koch C (2001). Computational modelling of visual attention. Neuroscience.

[CR31] Jovanovic L, Mamassian P (2020). Events are perceived earlier in peripheral vision. In Current Biology.

[CR32] Lleras A, Buetti S, Xu ZJ (2022). Incorporating the properties of peripheral vision into theories of visual search. Nature Reviews Psychology 2022 1:10.

[CR33] Luck SJ, Gaspelin N, Folk CL, Remington RW, Theeuwes J (2021). Progress toward resolving the attentional capture debate. Visual Cognition.

[CR34] Mathôt S, Schreij D, Theeuwes J (2012). OpenSesame: An open-source, graphical experiment builder for the social sciences. In Behavior Research Methods.

[CR35] McKee SP, Taylor DG (1984). Discrimination of time: Comparison of foveal and peripheral sensitivity. Journal of the Optical Society of America A.

[CR36] Meeter M, Olivers CNL (2006). Intertrial priming stemming from ambiguity: A new account of priming in visual search. Visual Cognition.

[CR37] Mulckhuyse M, Van Zoest W, Theeuwes J (2008). Capture of the eyes by relevant and irrelevant onsets. Experimental Brain Research.

[CR38] Müller H, Rabbitt P (1989). Reflexive and voluntary orienting of visual attention: Time course of activation and resistance to interruption. Journal of Experimental Psychology. Human Perception and Performance.

[CR39] Nyström M, Holmqvist K (2010). An adaptive algorithm for fixation, saccade, and glissade detection in eyetracking data. Behavior Research Methods.

[CR40] Peters RJ, Iyer A, Itti L, Koch C (2005). Components of bottom-up gaze allocation in natural images. Vision Research.

[CR41] Posner M (1980). Orienting of attention. Quarterly Journal of Experimental Psychology.

[CR42] Staugaard CF, Petersen A, Vangkilde S (2016). Eccentricity effects in vision and attention. Neuropsychologia.

[CR43] Strasburger H, Rentschler I, Jüttner M (2011). Peripheral vision and pattern recognition: A review. Journal of Vision.

[CR44] Theeuwes J (1991). Cross-dimensional perceptual selectivity. Perception & Psychophysics.

[CR45] Theeuwes J (1992). Perceptual selectivity for color and form. Perception & Psychophysics.

[CR46] Theeuwes J (2004). Top-down search strategies cannot override attentional capture. Psychonomic Bulletin and Review.

[CR47] Theeuwes J (2019). Goal-driven, stimulus-driven, and history-driven selection. Current Opinion in Psychology.

[CR48] Theeuwes J, Kramer AF, Hahn S, Irwin DE (1998). Our eyes do not always go where we want them to go: Capture of the eyes by new objects. Psychological Science.

[CR49] Theeuwes J, Kramer AF, Hahn S, Irwin DE, Zelinsky GJ (1999). Influence of attentional capture on oculomotor control. Journal of Experimental Psychology: Human Perception and Performance..

[CR50] Theeuwes J, De Vries GJ, Godijn R (2003). Attentional and oculomotor capture with static singletons. Perception and Psychophysics.

[CR51] van Heusden E, Donk M, Olivers CNL (2021). The dynamics of saliency-driven and goal-driven visual selection as a function of eccentricity. Journal of Vision.

[CR52] van Heusden E, van Zoest W, Donk M, Olivers CNL (2022). An attentional limbo: Saccades become momentarily non-selective in between saliency-driven and relevance-driven selection. Psychonomic Bulletin and Review.

[CR53] van Heusden E, Olivers CNL, Donk M (2023). The eyes prefer targets nearby fixation: Quantifying eccentricity-dependent attentional biases in oculomotor selection. Vision Research.

[CR54] van Opstal AJ, van Gisbergen JAM (1989). Scatter in the metrics of saccades and properties of the collicular motor map. Vision Research.

[CR55] van Zoest W, Donk M (2005). The effects of salience on saccadic target selection. Visual Cognition.

[CR56] Van Zoest W, Donk M (2004). Bottom-up and top-down control in visual search. Perception.

[CR57] Van Zoest W, Donk M (2008). Goal-driven modulation as a function of time in saccadic target selection. Quarterly Journal of Experimental Psychology.

[CR58] Van Zoest W, Donk M, Theeuwes J (2004). The role of stimulus-driven and goal-driven control in saccadic visual selection. Journal of Experimental Psychology: Human Perception and Performance.

[CR59] Wang B, Theeuwes J (2018). Statistical regularities modulate attentional capture independent of search strategy. Attention, Perception, and Psychophysics.

[CR60] Wang Z, Lleras A, Buetti S (2018). Parallel, exhaustive processing underlies logarithmic search functions: Visual search with cortical magnification. Psychonomic Bulletin and Review.

[CR61] Wolfe JM, Horowitz TS (2017). Five factors that guide attention in visual search. Nature Human Behaviour.

[CR62] Wolfe JM, O’Neill P, Bennett SC (1998). Why are there eccentricity effects in visual search?. Perception & Psychophysics.

[CR63] Wu SC, Remington RW (2003). Characteristics of covert and overt visual orienting: Evidence from attentional and oculomotor capture. Journal of Experimental Psychology: Human Perception and Performance.

[CR64] Wykowska A, Schubö A (2011). Irrelevant singletons in visual search do not capture attention but can produce nonspatial filtering costs. Journal of Cognitive Neuroscience.

[CR65] Yeshurun Y, Carrasco M (1998). Attention improves or impairs visual performance by enhancing spatial resolution. Nature.

[CR66] Zelinsky GJ (2008). A theory of eye movements during target acquisition. Psychological Review.

